# A Case of Foreign Bodies in the Stomach, with Treatment

**Published:** 1897-06

**Authors:** James S. Watson

**Affiliations:** Manor, Tex.


For the Texas Medical Journal.

### A CASE OF FOREIGN BODIES IN THE STOMACH, WITH TREATMENT.

BY JAMES S. WATSON, M. D., MANOR, TEX.

[Read before the Austin District Medical Society, March 21, 1897.]

ABOUT five years ago I was called to see a child three years old, who had swallowed some crushed glass fragments of a two drachm thin glass vial, with which it was playing, when it was crushed between its teeth, and some of the fragments swallowed, inflicting several small wounds in the mouth, and as low down the throat as I could see. I had read the mashed potato or so-called Vienna treatment, but in a child so young I could scarcely have given enough to insure much protection. At this juncture a treatment suggested itself which was at least new to me, so I will give it to you to-day.

Knowing the tenacity with which the harder oils adhere to glass, I determined to give them a trial.

Then I wanted one that might be given in large quantities without harm to the child, so I selected mutton tallow, which I found in the house, and gave one ounce melted, every two hours for twelve hours, then half that amount for twelve hours longer, my object being to push it entirely beyond the powers of digestion.

The effect was all that I could have asked. The surplus tallow was formed into balls similar to those formed in the old sweet oil treatment for gall stones, and the bit of glass formed the nucleus for some of these balls, and were thus rendered entirely harmless during the remainder of their course.

